# G-Quadruplex Structures in the Human Genome as Novel Therapeutic Targets

**DOI:** 10.3390/molecules181012368

**Published:** 2013-10-08

**Authors:** Joanna Bidzinska, Graziella Cimino-Reale, Nadia Zaffaroni, Marco Folini

**Affiliations:** Department of Experimental Oncology and Molecular Medicine, Fondazione IRCCS Istituto Nazionale dei Tumori, Via G. Amadeo 42, Milano 20133, Italy; E-Mails: joanna.bidzinska@istitutotumori.mi.it (J.B.); graziella.ciminoreale@istitutotumori.mi.it (G.C.-R.); nadia.zaffaroni@istitutotumori.mi.it (N.Z.)

**Keywords:** G-quadruplex, oncogene, small molecules, telomeres

## Abstract

G-quadruplexes are secondary structures that may form within guanine-rich nucleic acid sequences. Telomeres have received much attention in this regard since they can fold into several distinct intramolecular G-quadruplexes, leading to the rational design and development of G-quadruplex-stabilizing molecules. These ligands were shown to selectively exert an antiproliferative and chemosensitizing activity in *in vitro* and *in vivo* tumor models, without appreciably affecting normal cells. Such findings point to them as possible drug candidates for clinical applications. Other than in telomeres, G-quadruplexes may form at additional locations in the human genome, including gene promoters and untranslated regions. For instance, stabilization of G-quadruplex structures within the promoter of MYC, KIT, or KRAS resulted in the down-regulation of the corresponding oncogene either in gene reporter assays or in selected experimental models. In addition, the alternative splicing of a number of genes may be affected for a therapeutic benefit through the stabilization of G-quadruplexes located within pre-mRNAs. It is now emerging that G-quadruplex structures may act as key regulators of several biological processes. Consequently, they are considered as attractive targets for broad-spectrum anticancer therapies, and much effort is being made to develop a variety of ligands with improved G-quadruplex recognition properties. Quarfloxin, a fluoroquinolone derivative designed to target a G-quadruplex within ribosomal DNA and disrupt protein-DNA interactions, has entered clinical trials for different malignancies. This review will provide some hints on the role of G-quadruplex structures in biological processes and will evaluate their implications as novel therapeutic targets.

## 1. Introduction

**Figure 1:**
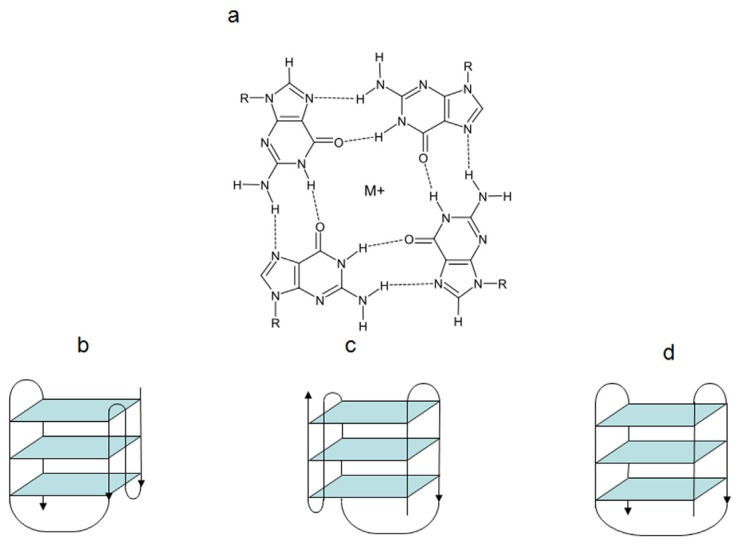
Schematic representation of a G-quartet arrengment (**a**) and of G4 structures with an intramolecular hybrid-type 1 (**b**), hybrid-type 2 (**c**) and basket-type (**d**) conformation. M^+^: alkali metal.

To date, many types of secondary non-B nucleic acids conformations have been identified. Among them are G-quadruplex (G4) structures which can form in guanine-rich nucleic acid sequences [1,2,3,4]. G4 structures are generated by a core of two or more π–π stacked G-quartets (Figure 1), which are stable planar arrangements of four guanine residues that are hydrogen-bonded via Hoogsteen pairings [1]. G4 structures are held together by intervening sequences of variable length that form single-stranded loops which are arranged on the exterior of the core [5]. These structures are further stabilized by monovalent cations (e.g., Na^+^, K^+^) that occupy the central cavities between the stacks, neutralizing the electrostatic repulsion of inwardly pointing guanine oxygens [6].

G4 structures may form under physiological conditions and show different topologies, the complexity of which depends basically on six variable parameters: (1) the oligonucleotide sequence; (2) the number of oligonucleotide strands (e.g., unimolecular, bimolecular, tetramolecular); (3) the directionality of strands (e.g., parallel, antiparallel, mixed); (4) the angles of the glycosidic bonds (e.g., *syn*, *anti*); (5) the size and type of intervening loops (e.g., diagonal loops, lateral loops and double chain reversal loops) and (6) environmental factors, such as the interacting alkali metals, the molecular crowding and the presence of binding ligands [6,7].

Bioinformatics analyses have revealed that ~400,000 putative G4 forming sequences (PQS) are present in the human genome [6,7]. These sequences consist of at least four runs of guanines (G-tracts), which usually contain at least three guanine residues (e.g., [G_≥3_N_x_G_≥3_N_x_G_≥3_]_≥4_, where N is any nitrogen base). Other than in human telomeric DNA, the PQS are frequently located within the promoter regions of oncogenes, suggesting that G4 structures may play a pivotal role in the control of a variety of cellular processes, including telomere maintenance, replication, transcription and translation [6,7].

Similar to proteins, the folded state of which creates specific druggable sites, the highly polymorphic nature of G4 conformations would make it possible, at least in principle, to rationally design small molecules able to selectively and differentially recognize and stabilize them. The most common types of G4 binding by specific ligands occurs *via* stacking onto or intercalation into the structure. In addition, owing to the structural complexity of G4, the groove and loop regions offer additional binding sites for selective recognition [7].

Although the physiological role of G4 structures still need to be intensively investigated, a growing body of evidence (mainly related to the role of G4 in the maintenance of telomere architecture/function) points towards such non-B DNA conformations as attractive targets for broad-spectrum anticancer therapies, and several ligands able to interact and stabilize G4 structures have been described during the past decades [8].

## 2. Targeting G4 Structures within Telomeres

Telomeres (from the Greek words *telos*- end and *meros*- part) are specialized DNA-protein structures located at the end of eukaryotic chromosomes. Human telomeric DNA consists of tandem repeated (TTAGGG)n sequences (3–15 kilobases) with a 150–200-nucleotide-long single-stranded terminus on the 3′-oriented strand (3′-overhang) [9]. Telomeres are bound directly or indirectly by a complex array of proteins, such as the six-protein complex shelterin, which includes the telomeric repeat binding factors 1 and 2 (TRF1, TRF2); the protection of telomeres (POT1); the transcriptional repressor/activator protein 1 (RAP1); the TRF1 interacting protein 2 (TIN2) and the POT1 and TIN2-organizing protein (TPP1) [9]. In addition to the shelterin complex, mammalian telomeres interact with other factors, including tankyrase 1 and 2, poly(ADP-ribose) polymerase 1 (PARP1), ataxia-telangiectasia mutated (ATM), ATM and Rad3-related (ATR), as well as general DNA replication and repair/recombination factors [9]. Such a nucleoprotein structure protects the chromosome ends (telomere capping function) from being recognized as DNA double strand breaks and, consequently, from being aberrantly processed by multiple pathways, such as ATM- and ATR-dependent DNA damage response (DDR), non-homologous end-joining (NHEJ), homologous recombination (HR) and resection, that may in turn result in genetic instability [9].

Telomeres uncapping may occur as a consequence of excessive telomere shortening, in that telomeres are presumably no longer able to form the protective higher order structure or bind sufficient amounts of shelterin factors and/or telomere-associated proteins, or when binding proteins, mainly TRF2 or POT1, are delocalized from telomeres [9]. 

In normal somatic cells, telomeres shorten with each round of cell division as a natural consequence of the inability of the DNA polymerase to completely replicate the chromosome ends (*i*.*e*., the end replication problem) [10]. Therefore, telomere erosion imposes in normal cells a finite number of cell divisions, thus representing a cell autonomous mechanism to prevent excessive telomere shortening and, as a consequence, genomic instability and malignant transformation.

Once a subset of telomeres become critically short (*i*.*e*., the Hayflick limit), cells cease to proliferate by entering a replicative senescence status. If inactivation of cell cycle checkpoints occurs, cells can escape replicative senescence and, after further telomere attrition, eventually enter a second growth arrest status (crisis). At this point, the occurrence of events like recombination and chromosome fusion may trigger genetic instability and often cell death. Occasionally, rare cells can emerge from this crisis and become immortalized by acquiring a telomere maintenance mechanism (TMM), an essential step during the transformation of most human cancer cells [10].

The most frequently reactivated TMM in human cancer is telomerase, an RNA-dependent DNA polymerase. The enzyme is composed of two core components: the hTR RNA subunit, which provides the template for the synthesis of telomeric DNA, and the TERT protein subunit, which possess a reverse transcriptase catalytic activity [11]. In addition, several other accessory proteins may regulate the enzyme biogenesis, the formation of functional holoenzyme complex and its cellular distribution [11].

Cancer cells that do not activate telomerase often rely on a recombination-based pathway for telomere maintenance known as alternative lengthening of telomere (ALT) mechanism [9]. The main features of ALT-positive cells are the presence of long and heterogeneous telomeres and of extrachromosomal linear and circular telomeric DNA fragments, the occurrence of spontaneous telomeric-localized DNA damage, as well as the presence of ALT-associated promielocytic leukemia bodies (APB), which are subnuclear bodies composed of telomeric DNA, shelterin factors and homologous recombination/DNA repair proteins [9]. 

Pieces of evidence suggest that cell-type specific mechanisms can favor the activation of one or the other TMM but the precise engine governing ALT is still to be disclosed in detail. Epigenetic alterations have been reported to influence which TMM is activated in specific cancer types [12] and, more recently, a mutational basis for the ALT activation, involving ATRX and DAXX genes, has been also evoked [13]. 

Since telomerase is activated in the vast majority of human cancer, but not in normal cells (except for germ cells, embryonic and stem cells) [14], the enzyme has been considered an excellent target for therapeutic interventions and several telomerase inhibitors have been described so far, thus contributing to validate the enzyme as a cancer-specific target [11]. However, telomerase and ALT may coexist in the same tumor [9]. Consequently, it is plausible that the use of telomerase inhibitors could exert a selection pressure leading to the emergence of sub-populations of ALT-positive cells refractory to telomerase inhibitors [9]. It has been recently reported that telomerase inhibition results in the acquisition of an ALT phenotype in a mouse model of T-cell lymphoma as well as in the overexpression/amplification of clue regulators of mitochondrial biology and function and of oxidative stress defense pathways [15].

In addition, the evidence that a significant fraction of solid tumors express ALT mechanisms instead of telomerase suggests that they will not likely be affected by any telomerase inhibitor [9]. However, due to the fragmentary knowledge concerning the molecular events governing ALT mechanism, inhibitors that specifically target this pathway have not been reported yet. 

In this regard, telomeric DNA has received much attention as the G-rich telomeric 3′-overhang can fold into tetraplexes. At the telomere level, G4 structures may play several biological roles [16]. Specifically, they may contribute to cap telomeres and pose a physical blockade for the access of telomerase to the chromosomes ends. Furthermore, G4 structures may also act as a barrier for the execution of the early steps of recombination required for ALT mechanisms [16]. In this context, investigations of the telomeric 3′-overhang architecture under physiological conditions have identified telomeric G4 as specific structural targets for the development of telomere-directed G4 therapies [17]. Therefore G4 stabilization is considered as an attractive strategy to fight cancer, independently of the operating TMM.

The human telomeric G4 structures have been investigated by physico-chemical approaches under physiologically relevant conditions and diverse topologies have been described [18], although a detailed characterization of human telomeric G4 structures is still urgently needed for a better structure-based rational drug design. Among the different topologies described thus far, the hybrid-type intramolecular conformations seem to be the major forms of human telomeric G4 in solution in the presence of K^+^ [18]. It has been reported that telomeric sequences can form *in vitro* two related hybrid-1 and hybrid-2 structures (Figure 1), that are in equilibrium in K^+^ solution, both containing three G-tetrads linked with mixed parallel/antiparallel-G-strands, which differ in their loop arrangements, strand orientations, tetrad arrangements and capping structures, thus providing specific drug binding sites [18]. It has been also suggested that such a structure polymorphism and dynamic equilibrium are intrinsic properties of human telomeric sequences and that the low energy barrier between the different forms may provide a means for specific protein recognition [18]. Several proof-of-concept experiments have confirmed G4 stabilization as a useful strategy for pharmacological intervention. In addition, the resolution of the crystal structure of human telomeric DNA in complex with different ligands [19,20] provided useful hints for the rational design of small molecules characterized by improved selectivity towards telomeric G4 structures. 

**Figure 2:**
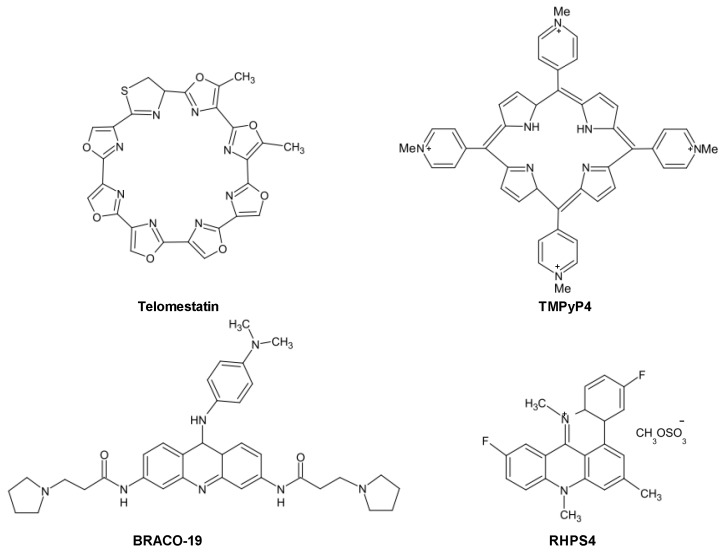
Chemical structures of historical telomeric G4-stabilizing agents.

The very first proof-of-principle was reported by Sun *et al*. [21] who showed that the stabilization of telomeric G4 structures by a 2,6-diamidoantraquinone resulted in the inhibition of telomerase activity *in vitro*. This encouraging result led to intensive screening for G4 stabilizing agents and, until now, several small molecules able to stabilize telomeric G4 structures have been described [22]. These compounds (Figure 2), belonging to a variety of chemical classes (e.g., cationic porphyrins, antraquinones, perylenes, fluoroquinolones (norfloxacin, ciprofloxacin), piperazines, pentacyclinacridinium salts, fluoroquinophenoxazines, ethidium derivatives, isoquinoline and benzylisoquinoline alkaloids, naphthalene diimides, bisquinolinium compounds, carbazole derivatives), share common features, such as the presence of a flat aromatic surface, of cationic charges as well as the ability to stack on or intercalate in targeted G4 structure.

Due to the great potential of the G4-based therapy there is a growing interest in the design and development of G4-stabilizing agents. In this context, comparative searches within the database of U.S. Food and Drug Administration-approved compounds and the literature to find molecules with the potential to bind G4 DNA have identified more than 750 telomerase inhibitors acting through G4 stabilization [23]. Subsequent evaluation of these compounds lead to the development of theoretical models able to discriminate the new G4 binders. Six compounds were predicted to bind to the human telomeric G4. Fluorescence resonance energy transfer (FRET) revealed that prochloroperazine, promazine, and chloropromazine stabilized the G4 structure. These compounds showed selectivity for the G4 structure over duplex sequence. Amitriptyline, imipramine, and loxapine were less efficient but also did bind to the G4 [23]. 

In addition, the combined use of molecular modeling, biophysical methods and click chemistry has generated a useful toolbox for the identification/design of optimized and more selective G4 targeting compounds. In this context, available databases of structural and biological data are used to perform high throughput *in silico* screenings for potential G4 interacting pharmacophores. For instance, virtual screenings represent a useful tool for the identification of promising candidates for their ability to interact with telomeric G4 [24]. Specifically, based on the assumption of a relationship between chemical structure and biological function, ligand- and structure-based approaches combined with tools for the prediction of the molecular properties have been used to select new pharmacophores on the basis of their similarity to known active drugs, and to discard compounds with unfavorable pharmacokinetic properties. Subsequently, the selected compounds have been submitted to docking simulation on all the characterized G4 conformations of human telomeric sequence and the resulting top ranked molecules were subsequently analyzed by conventional biophysical assays for their ability to bind and stabilize telomeric G4 structure [24]. By these methods, a new psoralen scaffold has been identified among an impressive number (~2.7 million) of compounds [24]. This evidence clearly underscores the importance and usefulness of such computational approaches, before going further into extremely expensive biological screenings. 

One of the most active and selective G4 ligand is the polyheteroaromatic molecule telomestatin, a natural compound derived from *Streptomyces anulatus*, with unique ability to stabilize G4 structures in the absence of monovalent cations. Telomestatin is the most potent telomerase inhibitor reported thus far [25,26]. The drug has been shown to greatly stabilize telomeric G4 and to preferentially bind to intramoleuclar G4, with a 70-fold higher selectivity for G4 over duplex DNA [25]. Telomestatin showed promising anticancer properties in several *in vitro* and *in vivo* models of human cancers, whereas it seems to not affect normal cells [27]. Other than causing telomerase inhibition, the drug may trigger telomere uncapping, as a consequence of the rapid delocalization of shelterin components as well as 3′-overhang degradation, eventually leading to an ATM-dependent DDR and cancer cell death [25]. The ligand showed also activity towards SV40-transformed ALT-positive human lung fibroblasts and caused delocalization of the Topoisomerase IIIα/Bloom helicase/TRF2 complex from telomeres, disrupted APB bodies and induced telomere-located DNA damage [28]. 

Another promising and deeply investigated telomeric G4 ligand is the pentacyclic acridine (3,11-difluoro-6,8,13-trimethyl-8H-quino[4,3,2,-kl]acridinium methosulfate, RHPS4. It is characterized by high selectivity for G4 DNA and inhibits telomerase activity in the submicromolar range [25]. The long-term exposure to subtoxic concentrations of RHPS4 resulted in a marked impairment of cancer cell growth accompanied by telomerase activity inhibition without appreciable telomere shortening. A deeper investigation of its mechanism of action revealed that the drug caused telomere dysfunctions, resulting in telomeric fusions, occurrence of polynucleated cells and telophase bridges [25].

Salvati *et al*. showed that RHPS4 is able to induce an ATR-dependent DDR at telomeres in melanoma cells as well as to cause replication stress [29]. When challenged on different human tumor xenografts in mice RHPS4 was shown to be very efficient in reducing tumor growth and metastasis compared to conventional antitumor drugs [30]. The compound showed also a high therapeutic index, in that it was well tolerated and did not cause general toxicity or body weight loss in mice, even though a marked but reversible hypotension was observed [30].

A large number of telomeric G4 ligands have been reported thus far, but remarkably few have been progressed to the point of being lead candidates in cancer drug discovery programs [31]. Among these ligands naphthalene diimide (NDI) represent promising scaffolds. Indeed, crystallographic analyses of the complexes between NDI and human telomeric DNA have provided a starting-point for rational optimization of these compounds. Modification of the NDI scaffold has been reported to increase the specificity for G4 over double-starnded DNA and to lead to better recognition between different G4 structures [31]. Recently, Doria *et al*. developed tri- and tetrasubstituted NDI composed of core tethered with quinone methides. These novel derivatives showed to selectively bind human telomeric G4 and to impair the growth of different human cancer cells following the induction of telomere dysfunctions and telomerase activity inhibition [32]. With the aim of enhancing telomeric G4 affinity and selectivity, the NDI BMSG-SH-3 (*N*,*N*′-bis(3-(4-methylpiperazin-1-yl)propylamino)-2,6-bis(3-(4-methylpiperazin-1-yl)propylamino)-1,2,5,8-naphthalenetetracarboxylic acid diimide), has been also recently designed by using molecular modeling on the basis of crystallographic data [31]. This compound showed sub-micromolar cell growth and telomerase inhibitory activity in a panel of pancreatic cancer cell lines. In addition, the compound demonstrated significant anti-tumor activity in an *in vivo* pancreatic cancer xenograft model [31]. Micco *et al*. recently reported the enhancement of the pharmacological properties of earlier NDI compounds using structure-based design. Crystal structures of three complexes with human telomeric intramolecular G4 demonstrated that two of the four strongly basic N-methylpiperazine groups can be replaced by less basic morpholine groups with no loss of intermolecular interactions in the grooves of the G4. The new compounds retain high affinity for human telomeric G4 and showed a 10-time increase in the cytotoxic activity when tested in pancreatic cancer cells. In addition, the lead compound triggered cell senescence and induced a dose-dependent modulation of the expression of genes involved in the DNA damage response (CDKN1A, DDIT3, GADD45A/G, PARP1, PPM1D) and in telomere maintenance (hPOT1) [33].

Carbazole derivatives able to stabilize telomeric G4 DNA have been designed and synthesized. Among them, 3,6-bis(1-methyl-4-vinylpyridinium)carbazole diiodide (BMVC) showed a potent inhibitory effect on telomerase activity. In a long-term setting, non-small cell lung cancer cells exposed to BMVC showed the typical hallmarks of senescence, including morphologic changes, senescence-associated beta-galactosidase activity, and decreased bromodeoxyuridine incorporation. Such a drug-dependent senescence phenotype was accompanied by progressive telomere shortening and induction of DNA damage. In addition, BMVC also impaired cell migration, colony-forming ability, and anchorage-independent growth and affected the tumorigenic potential of non small cell lung cancer xenografts *in vivo* [34].

Additional small molecules have been described for their selectivity toward G4 over double-stranded DNA, including pyridostatin, BMVC4 and phenanthroline derivatives. However, even though initially characterized as telomeric G4 ligands, several data indicate that they may interact with G4 structures located in different genomic loci thus suggesting that the reported antitumor effects may be the consequence of a more complex mechanism of action [35,36].

**Figure 3:**
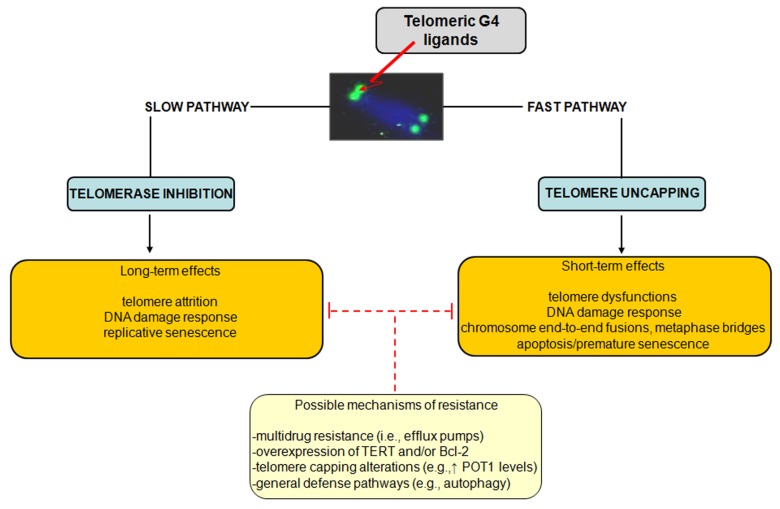
Schematic representation of the dual mechanism of action of telomeric G4 ligands. Possible resistance mechanisms that enable tumor cells to cope with telomeric G4-ligand-mediated detrimental effects have been also reported.

The influence of ligand-mediated G4 stabilization on cancer cell fate suggests that the observed responses may depend on several factors, including the type of cancer cell, the genetic background as well as the operating TMM. However, as schematically reported in Figure 3, a dual mechanism of action for telomeric G4 ligands has been consistently proved.

Specifically, G4-stabilizing agents may inhibit telomerase activity by locking the single-stranded telomere substrate into a G4 structure, resulting in long-term effects. Due to the inability of telomerase to extend a G4 folded telomeric substrate, G4-interacting agents were first evaluated as telomerase inhibitors and, in agreement with the initial paradigm for telomerase inhibition, long term exposure of human cancer cells to subtoxic doses of G4 ligands induces progressive telomere shortening and eventually replicative senescence. However, there is ample evidence that G4 ligands may also trigger short-term effects as a consequence of telomere uncapping and the subsequent rapid activation of a DDR, which overall may lead to any form of cell death or premature senescence. Finally, different G4 ligands have shown to be active *in vivo* as single agents and to act synergistically both *in vitro* and *in vivo* when combined with conventional (e.g., platinum compounds, taxanes and topoisomerase I inhibitors) or targeted (e.g., imatinib and PARP1 inhibitors) anticancer drugs [27].

Similarly to other therapeutic agents, it is plausible that even G4 ligands can undergo events of innate or acquired resistance. However, a few studies have reported to date resistance mechanisms dealing with the multidrug-resistance phenotype. In this context, it has been shown that the 2,4,6-triamino-1,3,5-triazine derivative, 12459, and the pyridodicarbxamide 360A may be recognized by efflux pumps, even though with low affinity [37].

Taking into account the dual mode of action of G4 ligands, attempts aimed at isolating cell sub-lines showing a resistant phenotype to G4 ligands have been pursued. Specifically, the JFA2 cell line obtained after the exposure of lung cancer cells to progressively increasing concentrations of 12459 showed to be resistant to the drug-mediated induction of senescence and cross-resistant to telomestatin [37]. The cell line showed also a resistant phenotype to short-term exposure to 12459 but not to the 3,6,9-trisubstituted acridine BRACO-19 and telomestatin, whereas no cross-resistance to conventional anticancer agents (e.g., doxorubicin, etoposide and topoisomerase I inhibitors) was observed [37]. This evidence highlights the occurrence of a selective mechanism of resistance to G4 ligands. By contrast, JFD cell sub-lines obtained after short-term exposure to high concentrations of 12459 showed cross-resistance to others triazine derivatives and to mitomycin C but not to BRACO-19 or telomestatin, indicating that such a resistance phenotype is restricted to 12459 and other triazine analogs as well as to DNA-damaging agents [37]. Strikingly, these resistant sub-lines were characterized by overexpression of TERT transcript, which was paralleled by enhanced telomerase activity, increased telomere length and presence of telomere capping alterations (e.g., increase expression of POT1) [37]. In this context, it should be taken into account that POT1 promotes the resolution of G4 structures *in vitro* acting in concert with Bloom helicase [38] and that its suppression by RNA interference leads to the loss of telomeric 3′-overhang, induces senescence, apoptosis and chromosomal instability [39]. Moreover, the overexpression of the antiapoptotic factor BCL2 has been reported to contribute to the resistance to apoptosis induction following the short-term exposure of A549 cancer cells to 12459. However, its overexpression does not affect the outcome observed (*i*.*e*., senescence induction) after the prolonged treatment with the G4 ligand [37].

Overall, these findings suggest that telomere length and status, the expression of telomerase components as well as the unbalance in the expression levels of factors involved in apoptosis may represent determinants of resistance to G4 ligands. However, more general cell defense pathways have been evoked as mechanisms activated by cells in their attempt to cope with the detrimental effects of telomeric G4 ligands. It has been recently reported that the exposure of melanoma cells to an anthracene-based G4 ligand resulted in the induction of autophagy, the inhibition of which resulted in the enhancement of the cytotoxic activity of the ligand. These data represented the first evidence of autophagy as a safeguard mechanism activated by cancer cells to counteract G4 ligand-mediated cellular stress [40].

An additional example of the relevance of G4 structures at telomeric level emerged from the discovery of the telomeric repeat-containing long non-coding RNA (TERRA) molecules, which originate following the transcription of telomeric DNA [41]. Due to the heterochromatic state of human telomeres and their low gene density, chromosome ends were considered for a long time as transcriptionally silent genomic loci. To date, telomeric transcripts have been reported in several organisms, including humans [41]. Mammalian TERRA molecules comprises (UUAGGG)_n_ sequences that are heterogeneous in length, ranging from approximately 100 bases up to more than 9 kilobases [41].

To date, several information has been gathered about TERRA biogenesis, which seems to be mainly regulated by the heterochromatic state of the telomeres [41,42], whereas the actual knowledge concerning the functions exerted by TERRA in human cells is almost negligible. Biochemical *in vitro* assays have suggested that TERRA may regulate telomere length by acting as a natural telomerase inhibitor, likely through competitive base-pairing to the template region of hTR [43]. TERRA seems also to interact with the catalytic subunit TERT of the enzyme independently of the RNA moiety [43]. In addition, TERRA nuclear localization and enrichment at telomeres indicate that the molecule might regulate several aspects of telomere structure and replication. In fact, it may interact with different telomere-associated proteins. Other than the shelterin components, TERRA has been indeed reported to interact with dyskerin, DNA-dependent protein kinase catalytic subunit, PARP1, RecQ helicase, topoisomerase I as well as heterogeneous ribonucleoproteins (hnRNPs) [44]. In particular, the hnRNPA1 protein, which specifically binds UAGGGA/U repeat-containing RNA molecules, might function as a molecular bridge between telomeric DNA and TERRA [45].

The hypothesis that TERRA can antagonize telomerase-dependent telomere maintenance along with the observation that telomerase-positive tumor cells have decreased TERRA levels compared to ALT-positive cancer cells [46], suggests that TERRA may have clinical relevance and could represent a novel target for the development of specific anticancer therapeutic interventions. It has been indeed recently reported that larynx and colon cancers as well as B-cell lymphoma have lower TERRA levels compared to their normal counterparts [47]. In addition, TERRA expression levels have been reported to inversely correlate with the presence of telomerase activity in astrocytoma and it is associated with an unfavorable prognosis [48]. Conversely, higher TERRA levels, which positively correlated with the proliferative index, were found in stomach, lung and colon cancer specimens compared to matched normal tissues [49]. 

Interestingly, TERRA molecules bear the same sequence as the 3′single-stranded telomeric overhang DNA. This evidence along with the observation that TERRA physically associate with hnRNPA1, which in turn is able to unwind G4 structures [50], has led to hypothesize that TERRA may fold into G4 structures. Biophysical assays have demonstrated that TERRA is able to form parallel G4 structures in Na^+^ or K^+^ solutions [51] as well as a hybrid-type parallel G4 in association with telomeric DNA [52]. In addition, it has been shown that the r(UAGGGUUAGGGU) TERRA sequence form a very compact structure consisting of two tandem stacked G4 structures each containing three G-tetrad layers [53]. Moreover, by means of synthetic TERRA-like probes functionalized with pyrene moieties at both the 5′ and 3′ ends, it has been demonstrated that human TERRA RNA is able to form a parallel G4 structures in living HeLa cells, thus providing evidence for the presence of G4 structures in native TERRA transcripts in human cells [54].

To date there are a few studies showing the stabilization of G4 within TERRA by small molecules, consequently we are still far away from the validation of TERRA as a novel target for G4-mediated stabilization. However, a screening of small molecules for their ability to discriminate between telomeric DNA and RNA G4 has been performed [55]. Specifically, data showed that the 2′-OH groups of the RNA represent an important constraint for ligand-mediated stabilization of G4 RNA [55], in that it may hinder the interaction between the ligand side-chains with the G4 loops. In fact, of four ligands tested (*i*.*e*., BRACO-19 and three different naphthalene diimide (NDI) derivatives) for their capability to interact with G4, only one NDI derivative showed a comparable binding affinity for both telomeric DNA and TERRA G4, whereas the BRACO19 and the other two NDIs preferentially interacted with telomeric DNA [55], suggesting that dissimilarities in G4-ligand binding affinities may be the result of different side-chain functionalities. 

In a recent work, the crystal structure of a 12-nucleotide long telomere RNA sequence complexed to a triazole-acridine ligand has been reported [56]. Specifically, a 2:2 molecular interaction resulted in the generation of a bimolecular G4. Specifically, the ligands are stacked on each other to form the boundary between the G4, whereas the loops—which play an active role in binding the acridine—are held in specific arrangements by multiple hydrogen bonding involving the 2′-hydroxyl groups [56]. Taken together, these data highlight the importance of assessing the effect of hydroxyl groups on the interaction between small molecule ligands and RNA-compared to DNA-based G4 when evaluating new scaffolds as selective G4 interacting compounds.

Overall, whereas synthetic oligonucleotides have been used to demonstrate G4 formation in TERRA sequence, proof of G4 structures in native TERRA transcripts *in vivo* and details of their functional significance are still in need of robust experimental support [3]. However, the available information open new landscapes for a deeper understanding of native TERRA G4 architecture, for the future design of agents able to selectively target it as well as for a detailed and unequivocal characterization of the biological responses resulting from the possible stabilization of G4 within TERRA in human cancer compared to normal cells.

## 3. Targeting G4 Structures in Gene Promoters and Messenger RNAs

**Figure 4:**
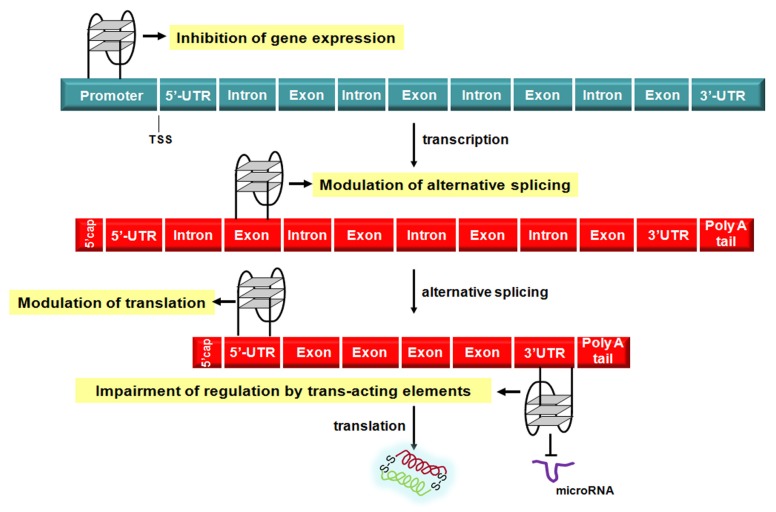
Schematic representation of G4-mediated regulation of the flow of genetic information. TSS: transcription start site; UTR: untranslated region.

Nowadays, there is compelling evidence that G4 structures play a prominent role in the modulation of the different steps of the flow of genetic information (Figure 4) [57]. The first and clearest evidence for a role of G4 structure in the regulation of gene transcription came from studies carried out on the v-myc avian myelocytomatosis viral oncogene, homolog (MYC), a transcription factor that regulates the expression of a variety of genes and is one of the most prevalent oncogenes found to be altered in human cancer, being deregulated in about 50% of tumors [58]. The transcriptional regulation of MYC is tightly controlled by a complex mechanism involving four promoters (P1–P4), different transcription start sites (TSS) and nuclease hypersensitive elements (NHE). In particular, the NHE III_1_, located just upstream the promoter P1 is responsible for the great majority of MYC transcriptional activity. It is composed of five consecutive runs of the sequence (G/A)G(G/A)AGGGGT that may form a G4 structure as well as an i-motif on the complementary, pyrimidine-rich strand [59]. As a consequence, the possibility to inhibit MYC transcription through G4 stabilization has been actively pursued in several human cancer models using specific small molecules [59].

The most studied MYC G4 stabilizer is the cationic porphyrin tetra-N-methylpyridyl porphyrin molecule (TMPyP4), which was firstly evaluated as a telomerase inhibitor due to its telomeric G4 stabilizing capabilities. It showed to potently stabilize MYC G4 structures in different cancer cell lines, resulting in a decrease of MYC gene transcription and protein expression and, consequently, to affect the expression of factors controlled by MYC, including TERT [60]. This evidence suggests that TMPyP4 acts with a dual mechanisms of action converging on telomerase-dependent telomere maintenance: (i) it prevents the access of telomerase to telomeres due to the stabilization of telomeric G4 and (ii) it decreases TERT expression levels owing to the stabilization of G4 within the promoter of MYC.

The regulation of MYC gene transcription is based on a fine interplay between transcription factors and dynamic consequence of transcriptionally induced negative superhelicity [61]. The elucidation of how such a complex transcriptional machinery works has provided the first-in-class example of a novel level of complexity in gene transcription as well as the very first evidence of the existence of G4-protein interactions in living cells. In particular, one of the main features to turn on/off MYC transcription deals with the formation and dissipation of G4/i-motif structures within MYC promoter, a process that is tightly controlled by nucleolin and NM23H2, which act concertedly with transcription-induced negative superhelicity [61].

Nucleolin is 100-kDa multifunctional nucleolar phosphoprotein that plays a role in a variety of cell functions. Because of its modular structure, it is able to interact with non-conventional forms of RNA and DNA. In particular, it has been reported that nucleolin selectively binds and stabilizes the parallel-stranded MYC G4, resulting in the inhibition of Sp1-induced MYC transcriptional activation [61]. In this context, the fluoroquinolone quarfloxin (CX-3543) has been demonstrated to indirectly affect MYC transcription, according to such a regulatory mechanism involving nucleolin. Specifically, the drug concentrates in the nucleolus where it binds and stabilizes a G4 within a ribosomal DNA resulting in the disruption of nucleolin/G4 complexes. This event causes the redistribution of nucleolin within the nucleoplasm where it eventually binds to the NHE III_1_, thus facilitating the formation and stabilization of the MYC G4, resulting in the prevention of gene transcription and induction of apoptosis [61].

NM23H2 is a member of the non-metastasis 23 family of proteins, able to bind the NHE III_1_ and promote the transcription of MYC [61]. The enzyme was found to bind the purine/pyrimidine-rich single stranded DNA but not the duplex. This evidence has led to propose that the protein may take advantage of the dynamic nature of the G4/i-motif structure of MYC and favor its unfolding [61]. Interestingly, it has been demonstrated that TMPyP4-mediated stabilization of G4 within MYC promoter impairs the binding and, consequently, the unwinding activity of NM23H2, thus contributing to the inhibition of MYC gene expression [61].

Additional ligands have been investigated for their ability to stabilize G4 within MYC promoter. For instance, the quindoline compound (a derivative of the natural product cryptolepine) has been shown to stabilize the G4 formed in the MYC promoter and to inhibit the expression of the oncogene in a hepatocellular carcinoma cell line [62]. In addition, the GQC-05 analogue of ellipticine was recently shown to bind with high affinity and selectivity the G4 structure within the NHE III_1_ region of MYC *in vitro* and caused down-regulation of MYC mRNA expression levels in a Burkitt’s lymphoma cell line [63].

A G-rich region located at −22 and −90 nucleotides from the TSS within the telomerase reverse transriptase (TERT) promoter contains 12 consecutive G-tracts of three or more guanine residues, embracing three Sp1 binding sites, and has the potential to fold into G4 conformations [64]. Biophysical investigations carried out on the full-length TERT G-rich sequence revealed that the core promoter adopts a tandem G4 structure composed of two intramolecular G4, a standard parallel and a hybrid-type G4 structure, with a 26-nucleotide long middle loop. A mutational study on the middle loop suggests that it forms a hairpin structure, which plays an important role for the stability of the G4 [64]. The formation of such a tandem G4 structure results in the sequestration of all Sp1 binding sites, thus preventing Sp1 binding to the TERT core promoter and, consequently, exerts an inhibitory effect on the promoter transcriptional activity [64]. In addition, the tandem G4 within TERT promoter may provide binding sites for selective recognition by G4-interacting agents. In this context, using a Taq polymerase arrest assay a similar decrease in the levels of the full-length product was observed with TMPyP4 or telomestatin [64], but not with TMPyP2, a positional isomer of TMPyP4 with low affinity for G4 structures. In addition, experimental data indicated that TMPyP4 binds between the two tandem TERT G4, whereas telomestatin preferentially recognizes the external tetrads of the tandem structure, although evidence has been provided that both drugs may interact with minor G4 that may form in the TERT G-rich sequence [64].

The documented capability of TMPyP4 and, more recently, of an NDI-based G4 ligand [32] to simultaneously stabilize G4 structures within telomeres and the promoters of MYC and TERT has led to propose that the extent of promiscuity of a given ligand for different G4 structures (*i*.*e*., multi-hit targeting) may represent an advantage for the therapeutic exploitation of G4 stabilizers. 

Recently, it has been reported that the perylene derivatives PM2 and PIPER were able to induce G4 formation both in telomeric DNA and TERT promoter region. Treatment of human lung cancer cells with these compounds resulted in the down-regulation of TERT mRNA expression levels and inhibition of telomerase activity. In addition, long-term treatment with sub-cytotoxic doses of these ligands led to telomere shortening, inhibition of cell proliferation and induction of senescence [65].

The v-kit Hardy-Zuckerman 4 feline sarcoma viral oncogene homolog (KIT) proto-oncogene encodes for a tyrosine kinase receptor that plays a pivotal role in cell survival, proliferation and differentiation [66]. Driving mutations of KIT receptor have been implicated in the pathogenesis of several cancers and efforts have been made for the identification of effective inhibitors of its kinase activity. Despite some benefits have been obtained following the use of inhibitors, including imatinib, sunitinib, dasatinib, certain oncogenic mutations may account for primary or secondary resistance [66].

A proposed approach for defeating the resistance to tyrosine kinase inhibitors deals with the selective stabilization of G4 structures that may form within the promoter region of the gene. Human KIT promoter contains indeed two conserved PQS, located at −12/−33 (KIT1) and −64/−83 (KIT2) nucleotides upstream of the TSS that may form unimolecular parallel G4 structures under physiological conditions [67,68]. A series of six 3,8,10-trisubstituted isoalloxazines has been evaluated for their selective binding to KIT G4 and all ligands tested showed a binding preference for KIT2 [69]. The effects on gene expression were evaluated using the ligands proved to induce the more stable G4 conformation. The ligands were able to markedly inhibit KIT expression suggesting that this class of compounds could be promising G4 ligands to target KIT-expressing cancer cells [69]. In addition, a bis-indole carboxamide showed a high level of stabilization for KIT2 [70]. However, the effects on gene expression levels were not assessed [70]. Moreover, two benzo[a]phenoxazines that showed high stability in the binding to the G4 in the KIT core promoter, with a preference for KIT2 over KIT1, were able to down-regulate KIT gene expression in human gastric carcinoma cells [71].

The concept of multi-hit targeting through G4 stabilization has been also highlighted for KIT. Specifically, it has been demonstrated that an NDI derivative potently inhibited the growth of a patient-derived gastrointestinal stromal tumor cell line as a consequence of its ability to markedly stabilize both telomeric and KIT G4 [72]. No significant changes in KIT expression levels were observed using BRACO-19 and TMPyP4, likely as a consequence of their lower G4 stabilizing capability compared to the NDI. This evidence suggests that a threshold level of G4 stabilization may be required to efficiently affect gene transcription.

An unexpected property of a triarylpyridine derivative, belonging to a class of molecules known for their selective interaction with G4 structures, deals with its ability to disrupt the structural integrity of the G-tetrads within KIT2 [73]. This event resulted in a marked increase in KIT expression levels when the molecule was administered to human cancer cells. This evidence suggests that the functional consequences of G4 interacting agents may depend on the specific mode of their interaction with the G4 structure, providing fundamental insights into the potential complexity of ligand/G4 interactions and how they might influence gene expression [73].

Another tyrosine kinase receptor that may undergo G4-dependent transcriptional regulation is the rearranged during transfection (RET) proto-oncogene, which is implicated in the initiation and progression of several human tumors [74] and represents a potential therapeutic target for the treatment of RET-associated cancers, such as thyroid cancers. A study of the transcriptional regulation of the RET proto-oncogene revealed that its promoter contains two GC boxes, located at −59 and −25 nucleotides from the TSS, which are essential for basal promoter activity [75]. In this region both DNA strands are extremely enriched in C- and G-containing sequences, which are very dynamic in nature and have the ability to adopt different non-B-DNA conformations [75]. Specifically, the polypurine-rich strand within this region consists of five consecutive runs of guanines, dealing with the general motif capable of forming intramolecular G4 [75].

The capability of such a G-rich strand to form G4 structures *in vitro* was investigated. DNA polymerase stop assay carried out on a wild-type RET template containing five runs of guanines (I, II, III, IV, and V) showed that in the presence of K^+^ a significant amount of arrested synthesis product appeared at the 3′-end of guanine repeat I. The minor stop product at the 3′-end of guanine repeat II indicated the formation of a G4 by guanine repeats II–V. This data suggests that the four consecutive guanine repeats I–IV in the G-rich strand of the RET promoter form the major G4 in the presence of K^+^ [75]. Comparative circular dichroism (CD) and dimethyl sulfate (DMS) footprinting studies have revealed that this structure is a very stable parallel-stranded intramolecular G4 made by three planar tetrads formed by four runs of guanine [75] and that TMPyP4 and telomestatin are able to efficiently stabilize it in presence of K^+^ and Na^+^. Of note, the concentration of K^+^ required to stabilize the RET G4 structures in the presence of either ligands was much lower than that required to stabilize it in their absence. This evidence suggests that TMPyP4 and telomestatin might act synergistically with K^+^ in stabilizing the tetraplex structures by binding them through external stacking at the ends of the G4 rather than through intercalation between the G-tetrads [75]. Recently, nuclear magnetic resonance analyses revealed that the core structure of RET G4 contains one G-tetrad with all *syn* G residues and two other with all *anti*-guanines. In addition, three double-chain reversal loops are also present, of which two are made of three GCG segments, whereas the remaining contains only one C. These loops interact with the core G-tetrads in a specific way that defines and stabilizes the overall RET G4 structure [76]. Such a specific alignment indicates that the overall G4 structure has a distinct pattern of grooves in comparison with the all parallel-stranded G4 within the promoter region of MYC suggesting that it could be an attractive target for pathway-specific drug design [76]. Finally, CD and DMS footprinting analyses, carried out on a synthetic oligomer, demonstrated that the C-rich strand of RET may fold intramolecularly to generate an i-motif, the stability of which is dependent on pH [75]. This additional non-B DNA conformation may provide an alternative opportunity for selective drug targeting (further details on i-motifs are provided in another paper in the present issue).

The activation of members of the rat sarcoma viral oncogene homolog (RAS) family of oncoproteins represents a key feature of malignant transformation for many cancers [77]. The three human RAS proteins (HRAS, NRAS and KRAS) function as GDP/GTP molecular switches for the control of several signaling networks involved in the regulation of cell proliferation, survival, differentiation and, more generally, gene expression [77]. Efforts to develop therapies to directly inhibit RAS oncoproteins have failed thus far. Conversely, progress has been made with inhibitors of RAS downstream signaling pathways, such as the RAF kinase inhibitor sorafeninb that has been approved for clinical use [77], and drugs aimed at blocking the mitogen-activated protein kinase/extracellular signal-regulated kinase kinase and the phosphatidylinositol-4,5-bisphosphate 3-kinase pathways, which are currently under clinical development [77]. Taking into account this scenario, drug-mediated G4 stabilization may hence represent a novel alternative for silencing the RAS signaling pathway. In this context, an NHE upstream from the major TSS has been identified in the human KRAS promoter. This polypurine/polypyrimidine sequence, located between −327 and −296 nucleotides, presents consecutive runs of guanines that may assume a parallel-stranded intramolecular G4 structure [78] able to interact with at least three nuclear proteins, of which hnRNPA1 displays G4 unwinding activity [79]. The folding topology of this structure is assumed to be similar to that of MYC. In addition, it has been shown that TMPyP4 can stack to the external G-tetrad of this G4 and that drug-mediated G4 stabilization resulted in the inhibition of promoter activity in a gene reporter assay [78]. Recently, the synthesis and G4 thermal stabilisation effects of a series of indolo[3,2-*b*]quinolines mono-, di-, and trisubstituted with basic side chains have been reported. Specifically, among these derivatives, the trisubstituted compounds 3d and 4d (bearing a 7-(aminoalkyl)carboxylate side chain) stand out as the most promising compounds showing high G4 thermal stabilisation and a 10-fold selectivity for G4 over duplex DNA. Morevoer, compounds 3d and 4d also decreased KRAS protein expression levels in colon cancer cells [80].

Two G-rich elements, *hras1* and *hras2* that fold respectively into an antiparallel and a parallel G4, have been identified within HRAS promoter [81], the activity of which was inhibited by the G4-ligand guanidium phthalocyanine in a gene reporter assay. In addition, the stability of such a G4 structure seems to be affected by the MYC-associated zinc finger protein (MAZ) transcription factor, which shows an unexpected G4 unwinding activity and acts as an HRAS transcriptional activator by binding to the unfolded conformation of *hras1* and *hras2* G4-forming elements. This evidence was further corroborated by a decoy strategy based on the use of HRAS G4 mimicking oligomers. Such G4-decoys repressed HRAS transcription, likely as a consequence of MAZ sequestration, and caused a strong cell growth inhibition and apoptosis induction in bladder cancer cells [81].

Programmed cell death is a well-orchestrated process regulated by multiple pro-apoptotic and anti-apoptotic genes. Deficiencies in the apoptotic pathway are a hallmark of cancer responsible for the limited effectiveness of anticancer drugs [82]. The B-cell CLL/lymphoma 2 (BCL2) is an anti-apoptotic factor which is overexpressed in several human cancers [82], where it contributes to resistance to treatment by conventional anticancer approaches. Small molecule inhibitors and peptides (*i*.*e*., BH3 mimetics) as well as antisense and gene therapy strategies have been widely used to counteract the antiapoptotic activity of BCL2 in different cancer models [83].

A 39 base-pair GC-rich region (Pu39) upstream of one of the two promoters of BCL2 gene has been shown to be critically involved in the regulation of gene expression [83]. Such a guanine-rich DNA strand has the potential to form multiple intramolecular G4 structures *in vitro*. Indeed, three separate DNA sequences within this region, which may form individual G4 structures, were characterized [84]. The most stable G4 forms within the middle four runs of guanines, in that it requires the least amount of K^+^ for stabilization in comparison with the 5′- and 3′-end runs [84]. The ability of TMPyP4 as well as of the core-modified porphyrin analogue, 5,10,15,20-[tetra-(N-methyl-3-pyridyl)]-26,28-diselenasapphyrin chloride (Se2SAP) and telomestatin to selectively interact and stabilize the three G4 structures was investigated [84]. The results revealed that TMPyP4 and Se2SAP did have a structural selectiveness for the different G4, whereas telomestatin had the ability to interact quite strongly with all sequences. These results suggest the possibility to selectively target, through the use of different G4 interactive molecules, the three constitutive G4 within the BCL2 promoter, which may result in different biological outcomes. Recently, three quindoline derivatives (SYUIQ-01, SYUIQ-F05 and SYUIQ-FM05) were tested for their ability to interact with G-rich sequences located within BCL2 promoter. Specifically, all tested ligands showed good binding selectivity for G4 DNA in surface plasmon resonance assay, even though compound SYUIQ-FM05 was the most selective molecule. In addition, the exposure of acute promyelocytic leukemia cells to SYUIQ-FM05 resulted in a pronounced inhibition of BCL2 gene expression, cell growth arrest and induction of programmed cell death [85]. 

The hypoxia inducible factor 1 alpha (HIF1A) is a transcription factor that plays a critical role in mediating cellular responses to hypoxic conditions [86]. HIF1A protein, generally absent in most normal tissues, is overexpressed in many human cancers and represents an attractive target for therapeutic interventions [86]. A polypurine/polypyrimidine tract, located between −65 and −85 nucleotides upstream of the TSS has been identified within the proximal promoter region of HIF1A. The importance of this tract in the regulation of gene transcription has been confirmed by the observation that mutagenesis of this region results in lower basal HIF1A expression [87]. Electrophoretic mobility shift assay, CD, Taq polymerase stop assay, and DMS footprinting analyses performed on synthetic oligomers have revealed that this polypurine/polypyrimidine tract may form an intramolecular parallel-stranded G4 structure in the presence of K^+^ [87]. Furthermore, DNA polymerase stop assay showed that TMPyP4 and telomestatin were capable of binding to and stabilizing such a G4, whereas TMPyP2 did not. Recently, the naphthalene derivative CL67 has been reported to selectively interact with the HIF1A G4 and to cause down-regulation of HIF1A expression levels in renal cancer and osteosarcoma cells [88]. Whether this effect was related to the specific stabilization of HIF1A G4 or, alternatively, of other tetraplexes forming in PQS within the HIF1A pathway, still remains to be ascertained [88]. 

Additional examples of gene promoters (e.g., MYB, VEGF, PDGFA, PDFGR-β) endowed with the ability to form G4 structures under physiological conditions and that may represent suitable targets for small molecule-dependent G4 stabilization have been recently identified [89].

One of the most promising G4 selective molecules is pyridostatin, which it has been recently shown to promote growth arrest in human cancer cells by inducing replication- and transcription-dependent DNA damage [90]. Specifically, chromatin immunoprecipitation sequence analysis of the DNA damage marker γH2AX provided the genome-wide distribution of pyridostatin-induced sites of damage, and revealed that the compound was able to target gene bodies containing clusters of PQS. Since local DNA damage within a genomic locus can trigger transcriptional inhibition in *cis*, the authors investigated whether pyridostatin affected the mRNA levels for MYC and the top ten γH2AX-positive genes containing the highest PQS densities identified in the previous analyses. In particular, they found that whereas the expression levels of control genes were not affected by pyridostatin treatment, all the γH2AX-positive targets analyzed, of which SRC (Schmidt-Ruppin A-2 viral oncogene homolog sarcoma) was the most strongly affected gene, were down-regulated after 8 h of drug treatment [90]. The SRC family kinases are the largest family of non-receptor tyrosine kinases involved in several cell processes. SRC is one of the oldest oncogene identified as well as one of deeply studied targets for anticancer therapy [91].

CD and NMR spectroscopy analyses showed that 23 out of 25 PQS within SRC gene body were able to adopt a stable G4 conformation and that pyridostatin selectively interacted with the G-quartet through a stacking mode, thus acting independently of G4 polymorphism [90]. In addition, pyridostatin-mediated down-modulation of SRC expression levels resulted in a marked impairment the *in vitro* motility of breast cancer cells as assessed by a wound healing assay. Moreover, a caged pyridostatin, obtained by introducing a photolabile aromatic group to the core of the molecule, showed to efficiently down-regulate SRC expression levels in SV40-transformed MRC-5 fibroblasts upon UV irradiation [92]. These results highlighted the possibility to obtain a spatiotemporal regulation of gene expression and paved the way for future consideration of G4-based photodynamic therapies [92].

The observation that only up to 2% of the all transcribed RNA molecules are translated into proteins has contributed to highlight that the vast majority of RNA species produced within a cell are actually the cornerstone of the post-transcriptional regulation of gene expression [93]. In fact, other than mRNA processing events (e.g., capping, splicing and polyadenalytion), active transport, stability and translation, additional mechanisms to control RNA transcription/translation include *trans*-acting RNA species, among which microRNAs play a paramount role [93], as well as *cis*-acting regulatory factors, usually represented by highly ordered RNA structures that may form in either the 5′- or 3′-untranslated regions (UTR) [3,93]. In this context, analyses based on computational approaches have revealed that a huge number of proteins coding RNAs are characterized by PQS located in their 5′- and 3′-UTR [93]. This evidence suggests that the formation of G4 structures within specific region of mRNAs may represent an additional as well as tunable *cis*-acting device by which RNAs exert their control on gene expression (Figure 4). Specifically, several experimental findings support a pivotal role of G4 structures forming within 5′-UTR and associated trans-acting factors in both cap-dependent and -independent (*i*.*e*., internal ribosome entry sites) regulation of the translation of protein coding genes [3].

In principle, G4 structures forming within RNAs should be more thermodynamically fostered than their DNA counterpart, mainly because of the single-stranded nature of RNA, which is not subject to competition for the hybridization to a complementary strand. In addition, G4 RNA may be more stable compared to G4 DNA as the connecting loops are held in particular conformations by multiple hydrogen bonding involving 2′-C hydroxyl groups of the ribose [3,55,56]. 

The early evidence of a G4 forming within an RNA molecule dates back to 1994, when an intramolecular G4 was evidenced *in vitro* in the 3′-UTR of the mRNA encoding for the insulin-like growth factor II [94]. Successively, several additional studies have tried to address the formation of G4 within RNA and to elucidate their role in biological systems. For instance, a highly conserved PQS has been identified within the 5′-UTR of human NRAS proto-oncogene, able to form a stable intramolecular G4 structure, even in the absence of K^+^ [95]. Using a reporter gene assay in a cell-free translation system it has been demonstrated that such a G4 RNA was able to affect the cap-dependent protein translation [95], a finding that was successively corroborated by the observation of the inhibition of protein translation in living eukaryotic cells following the formation of a G4 structure within the 5′-UTR of the mRNA encoding for human Zic-1 zinc-finger protein [96] and, more recently, within the 5′-UTR region of TRF2 mRNA [97]. Additional genes the cap-dependent translation of which may be modulated by the formation of G4 structures in their 5′-UTR include the matrix metalloproteinase MT3-MMP, the estrogen receptor ESR1, the anti-apoptotic BCL2 and the α–secretase ADAM10 [3]. Whereas the role of G4 structures in the cap-dependent translation has been mainly associated to the repression of gene expression, gene reporter assays have shown that cap-independent translation of human fibroblast growth factor 2 and vascular endothelial growth factor is favored by G4 formation [3]. 

Furthermore, evidence indicates a role of G4 forming sequence in the control of the alternative splicing, a regulated process by which multiple mRNA variants are produced from a single gene. In this context, it has been reported that the intron 6 of TERT pre-mRNA contains several G-tracts that can fold into a G4, which in turn may be stabilized by the triazine derivative 12459 [98]. Interestingly, short-term exposure of lung adenocarcinoma cells to compound 12459 resulted in the down-regulation of telomerase activity as a consequence of a shift in the splicing pattern toward the production of a catalytically inactive form of TERT [98]. 

Recently, G4 structures have been demonstrated to form within the 3′-UTR of the low-density lipoprotein receptor-related protein 5 (LRP5) and the fragile X-related mental retardation autosomal homolog 1 (FXR1) genes [99]. Specifically, folding into such a G4 results in the increase of the efficiency of alternative polyadenylation sites and leads to the expression of shorter transcripts or, in the case of FXR-1 gene, in the interference with the microRNA-dependent negative regulation of gene expression [99].

The pivotal role of G4 structures in the control of mRNA translation as well as in any other aspect of RNA metabolism (e.g., alternative splicing) has been also pointed out by the identification of RNA associated factors, such as RHAU, DHX9, CBF-A and hnRNPA2 that, similarly to DNA helicases, show G4 unwinding/destabilizing activity [3].

Although many efforts are still to be made to rigorously validate RNA G4 as drug targets for therapeutic intervention, the evidence that small molecule G4 binding ligands (including pyridine-2,6-bisquinolino dicarboxamides, bisquinolinium compounds and alkyl derivatives of cationic porphyrin) can selectively target RNA G4 resulting in the translational repression of target genes opens up new avenues in the design of G4 RNA specific drug candidates [3].

## 4. Conclusions

The recognition of the biological significance of G4 DNA has put a new wave of interest in the search and development of G4 interactive compounds. Targeting such a secondary DNA structures has represented an entirely novel approach to anticancer drug design and development during the last years. Nonetheless, there are still several hurdles that need to be brought down before these peculiar compounds will take part of the currently available armamentarium of anticancer agents.

The high prevalence of G4 in the human genome may raise concerns about the specificity of G4-stabilising agents, even if the great structural variability of G4 structures stands for their potential selective recognition. Recent works have highlighted the conformational heterogeneity of human telomeric G4 structures depending on the experimental conditions [18,100]. As a consequence, the detailed knowledge of a given G4 structures represents an essential starting point to overcome the problem related to G4 ligand selectivity, even if attention should be paid when comparing structure information obtained through different biophysical methods and under variable experimental conditions [100]. The crystal structure of the complex made of a tetra-substituted NDI with human telomeric G4 has been reported [19]. Interestingly, after ligand addition the telomeric G4 topology did not change compared to the drug-free structure. Specifically, it persisted as parallel-stranded with external double-chain-reversal propeller loops with the ligand stacking onto G-tetrad surface as well as into TTA loops. This evidence has revealed the peculiar binding mode of such a compound, allowing for future scaffold optimization in terms of selectivity and enhanced affinity [19], that may be achieved by introducing onto the G4 ligand core structure (*i*.*e*., aromatic surface) specific side chains able to interact with G4 grooves and loops. 

Another point that still need to be addressed regards the *in vivo* existence of G4 structures, which has been a matter of debate for decades. The possible presence of G4 structures *in vivo* has been indirectly pointed out by the identification of a variety of proteins able to stabilize or promote the formation of as well as to destabilize or unwind the tetraplex DNA [101]. In this context, RNA selection methodology was used to demonstrate that the FMRP (fragile X mental retardation protein) binds intramolecular G-quartets in target mRNAs, which suggested that G-quartets serve as physiologically relevant targets for FMRP [101,102]. Moreover, the regulation of FMRP expression was demonstrated to be dependent on the binding of the protein to its own mRNA through a G4 structure forming within the coding region [3]. Furthermore, evidence that defects in G4 metabolism may be connected with human genetic diseases has been provided [103].

However, the proof of G4 existence *in vivo* was obtained in ciliates by an RNAi-mediated approach showing that telomere end-binding proteins alpha and beta cooperate to control the formation of an antiparallel G4 DNA structure at telomeres *in vivo* [104,105]. In addition, an electron microscopy-based assay showed that G4 DNA formed within G-rich regions in transcribed plasmid genomes of *Escherichia coli* [106]. Morevoer, by using a click chemistry approach, a pyridostatin analogue (pyridostatin-α) was used to demonstrate the *in vivo* formation of G4 structures in a human osteosarcoma cell line stably expressing the nuclear isoform of human DNA helicase Pif1 (hPif1α) fused to a green-fluorescent protein. By the identification of overlapping signals between the ligand and the enzyme, results revealed a considerable overlap between the labeled small molecule and GFP-hPif1α foci, suggesting that the ligand and hPif1 target overlapping genomic structures in human cells. This study provided evidence also for the existence of pre-folded G4 structures at non-telomeric locations within human genomic DNA and suggested a role for hPif1 in the resolution of these structures *in vivo* [90]. More recently, clues for G4 formation in a cell cycle-dependent manner in the genome of mammalian cells have been provided [107]. The very recent development of G4 structure-directed antibodies that allow to quantitatively visualizing G4 structures in human cells has undoubtedly represented a step of paramount importance in the G4 research field [107,108]. Interestingly, a five-time increase in the fluorescent signals was observed during the S phase of the cell cycle, when the double-stranded DNA undergoes melting [107]. However, other than at telomeric level, most of the fluorescent signal was detected throughout the genome, suggesting that such a recently developed tool is still far from the possibility to discriminate between G4 structures that may form at different genomic loci. In addition, the use of an engineered antibody able to enrich for DNA containing G4 structures coupled to a deep sequencing analysis revealed that plasmacytoma variant translocation 1 gene contains G4 structures within the transcribed region and that the expression level of the gene resulted strongly up-modulated in cancer cell treated with pyridostatin [108].

Since telomeres, proto-oncogene promoters and mRNAs are present not only in cancer cells but also in normal cells, an additional issue that needs to be addressed for G4 stabilizing agents deals with their therapeutic index. Nevertheless, it cannot be excluded that differences in promoter epigenetic modifications, cell proliferation-dependent transcriptional activity, presence of single nucleotide polymorphysms as well as protein composition at telomere could account for a lower susceptibility to G4-interacting agents of normal compared to cancer cells. Unfortunately, to the best of our knowledge, comparative evaluations of the biological activity of ligands based on their selectivity for G4 structures forming within promoters or RNA molecules in normal *vs*. tumor cells have not been reported yet. However, evidence showing that telomeric G4 ligands selectively impair the growth of cancer cells without affecting the viability of normal cells (mainly fibroblasts) points to these molecules as possible drug candidates for future clinical applications [27]. This evidence gains further support by the marked antitumor activity showed by some of these compounds in different *in vivo* models with no signs of general toxicity or body weight loss [30]. Nevertheless, it is worth to underline that following the evaluation of the *in vivo* activity of telomeric G4 ligands, the differences in telomere biology between humans and mice should be taken into careful consideration.

Although different ligands have been documented to exert at a preclinical level good antiproliferative and antitumor effect, as a consequence of the stabilization of G4 structures, none of these compounds is currently under clinical development. The only exception is quarfloxin, a G4 stabilizer that entered phase I/II clinical trials for the evaluation of safety, tolerability and efficacy in patients with solid tumors (http://clinicaltrials.gov/).

In spite of the high number of ongoing studies on G4 ligands, there is a need for careful consideration of the experimental conditions and their unification/standardization to enable comparison of data and to make proper conclusions in terms of characterization of the biological responses and the observed phenotypes (e.g., resistance phenomena), as a function of the different tumor models. Once these aspects will be properly faced, such class of molecules will likely turn out into effective anticancer drugs.
